# Measuring discrepancies between simple medullary and synchronous medullary/papillary thyroid carcinomas: a comparative cross-sectional study

**DOI:** 10.3389/fendo.2023.1301200

**Published:** 2024-01-22

**Authors:** Daqi Zhang, Mingyu Yang, Francesco Frattini, Andrea Cestari, Kunlin Li, Hongbo Wang, Hao Chi, Chengqiu Sui, Kecheng Bai, Dongyuan Lan, Gianlorenzo Dionigi, Hui Sun

**Affiliations:** ^1^ Division of Thyroid Surgery, China-Japan Union Hospital of Jilin University, Jilin Provincial Key Laboratory of Surgical Translational Medicine, Jilin Provincial Precision Medicine Laboratory of Molecular Biology and Translational Medicine on Differentiated Thyroid Carcinoma, Changchun, Jilin, China; ^2^ Division of Surgery, Istituto Auxologico Italiano Istituto di Ricovero e Cura a Carattere Scientifico (IRCCS), Milan, Italy; ^3^ Department of Pathophysiology and Transplantation, University of Milan, Milan, Italy

**Keywords:** thyroid gland, medullary thyroid carcinoma, papillary thyroid carcinoma, synchronous lesions, calcitonin, CEA, pathology, follow-up

## Abstract

**Objective:**

To study the clinicopathological characteristics of patients with synchronous medullary and papillary thyroid carcinomas.

**Methods:**

The clinical data of patients with medullary thyroid carcinoma (MTC) operated in our hospital (Department of Thyroid Surgery, China-Japan Union Hospital, Jilin University) from February 2009 to February 2023 were evaluated using an analytical review approach. They were divided into an observation group (patients with synchronous MTC and papillary thyroid carcinoma PTC) and a control group (simple MTC) according to whether the clinical data were associated with MTC, in order to compare the clinical features, pathological types, stage characteristics and molecular biology characteristics of the two groups and to investigate the follow-up of the two groups.

**Results:**

The study included 122 MTC, 30 with synchronous MTC/PTC and 92 simple MTC. When the data were compared, the sex ratio, preoperative calcitonin level, preoperative CEA level, presence of calcifications in the MTC lesions, surgical methods, number of MTC lesions, presence of nodular goitre and presence of thyroiditis were higher in the observation group than in the control group. There was a significant difference between the groups when the MTC lesion diameter was ≤1cm in terms of preoperative CEA value (P<0.05); when the MTC lesion diameter was >1cm, there was a statistical difference between the two groups in terms of preoperative Ctn value (P<0.05). Type III was significantly different from the simple group, while type IV was more similar to the simple group. The preoperative serum Ctn value was positively correlated with maximum tumour diameter in both groups, although the correlation was stronger in the easy group. Preoperative CEA was positively correlated with maximum tumour diameter in both groups, with a stronger correlation in the combination group. Preoperative Ctn and CEA were positively correlated with lymph node metastasis in the simple group, whereas there was no apparent correlation with lymph node metastasis in the combination group. The cut-off value of preoperative serum Ctn for cervical lymph node metastases in the simple group was 39.2pg/ml and for lateral cervical lymph node metastases 195.5pg/ml. The cut-off value of preoperative serum Ctn for cervical lymph node metastases in the combination group was 60.79pg/ml and for lateral cervical lymph node metastases 152.6pg/ml. In the simple group, prognosis was significantly worse in the progression group (P<0.001), with no statistical difference between the remission and stable groups. In the combination group, the prognosis of the progression and stable groups was significantly worse than that of the remission group (P<0.001), with no statistical difference between the progression and stable groups.

**Conclusion:**

In patients with synchronous medullary and papillary thyroid carcinomas, preoperative Ctn and CEA levels, calcifications, solitary lesions, combined goitre or thyroiditis differ significantly from simple MTC. Therefore, clinical management should pay attention to the above factors and early risk screening should be performed to improve prognosis as much as possible.

## Introduction

Papillary thyroid carcinoma (PTC) accounts for more than 90% of all thyroid carcinomas (1). PTC arises from follicular epithelial cells derived from the endoderm and grows slowly.

Medullary thyroid carcinoma (MTC) accounts for 5 to 10% of all thyroid carcinomas. MTC arises from parafollicular C cells derived from the ectodermal neural crest and has an insidious onset and a higher malignancy grade (1, 2).

The histopathological features and prognoses of PTC and MTC are different, and their co-occurrence is very rare, accounting for less than 1% of thyroid tumours (1–6).

In our previous study, we presented an exceptional number of synchronous MTC/PTC in the same thyroid gland (2). Few studies compared the clinicopathological features of simple MTC and synchronous MTC/PTC (1–6).

Therefore, the aim of this second part of the study was to compare the clinicopathological features and follow-up of patients with simple MTC and synchronous MTC/PTC by analysing the clinical data of 122 consecutive MTC patients admitted to our hospital between February 2009 and February 2023.

The reports are as follows.

## Methods

### Protocol and time frame

This study retrospectively examined patients who were consecutively diagnosed with MTC between February 2009 and February 2023. It was a secondary research using previously collected and presented data (2).

### Location

Department of Thyroid Surgery, China-Japan Union Hospital, Jilin University, PR China.

### Ethics

The study was approved by the hospital ethics committee (No. 20220804013). As this was a retrospective study, informed consent was waived.

### Data sources and search strategy

The Department of Thyroid Surgery database is a statutory registry to record all newly diagnosed thyroid cancer cases. It is under the auspices of the China-Japan Union Hospital of Jilin University. Data were collected using a pre-designed data collection form.

### Selection criteria

All patients underwent surgical resection. MTC diagnosis was confirmed by histopathology. Patients who were pathologically diagnosed with primary MTC were eligible for inclusion in the study. The inclusion criteria were:

A. Combination group: ①First thyroid surgery in our department; ②Paraffin pathology after surgery with definitive diagnosis of synchronous MTC/PTC; ③Patients with complete medical records and follow-up data.

B. Simple group: ①First thyroid surgery in our department; ②Paraffin pathology after surgery with definitive diagnosis of MTC, not combinable with other tumour types; ③Patients with complete original medical record and follow-up data.

Exclusion criteria: ①other pathological thyroid carcinomas, benign tumours, other malignant tumours of the head and neck, parathyroid disease; ②first surgery in another hospital; ③incomplete or lost data.

### Pathological evaluation

Histopathological evaluation of all cases was done by a pathologist. The histopathological MTC diagnosis of the gross specimen was documented and compared. The histopathological findings were stained with H&E stains, Congo red and periodic acid-Schiff and documented as per the standard protocol. Histopathological examination of the gross specimen was taken as the final diagnosis. PTC and MTC. PTC and MTC were defined according to the ATA and Joint Committee of Cancer (AJCC, 8th edition) guidelines (7, 8).

Sinchronic MTC/PTC tumours were defined according to Sung CT and Sadow P as tumours consisting of two histologically distinct neoplasms arising in the same thyroid gland, i.e. one MTC is accompanied by a second tumour component arising from the follicular epithelial cells (9, 10).

### Classification

In our previous report, we classified synchronous MTC/PTC into four types (2):

Type I. True mixed MTC/PTC, i.e. MTC and PTC are closely intertwined; this is also referred to as true mixed medullary-follicular carcinoma (2).
Type II. Collision MTC/PTC, i.e. tumours consisting of two histologically distinct neoplasms that meet at the same site, invade each other and appear as a single mass in the thyroid gland, i.e. MTC and PTC merge (2).
Type III. Synchronous, anatomically separate tumours in the same thyroid lobe separated by non-tumour thyroid parenchyma (2).
Type IV. synchronous tumours occurring in separate anatomical lobes or in the isthmus (2).

### Preoperative imaging

Ultrasonography (US) was performed using high-frequency probes (at least 13 MHz). All thyroid nodules were assessed by a physician with approximately 10 years of experience in thyroid medicine. The optimal settings were always made in the same order (gain, field of view, magnification) to obtain ideal measurements of all thyroid nodules examined. The nodule was assessed by measuring the long axis on the US scan on greyscale images.

### TNM staging

Staging was done in all cases by US examination and in histopathological cases where cervical lymph node dissection had been performed (7, 8). Thyroid cancer is staged according to the most advanced PTC or MTC.

### Endpoints

Relevant clinical histories and radiological examinations were performed in all MTC patients. Data on clinical and pathological features were collected and compared between the two groups, including sex, age, body mass index (BMI), clinical symptoms, laboratory tests, imaging studies, surgical data, pathological results, morbidity and follow-up prognosis.

### Follow-up

All patients were followed up at the Department of Thyroid Surgery, China-Japan Union Hospital, Jilin University. Follow-up included measurements of Ctn, thyroglobulin, TSH and free thyroxine in all patients (1) on suppressive therapy and annual ultrasound examination of the neck (7). Diagnostic whole-body scans and determination of thyroglobulin after discontinuation of thyroxine were performed 9-12 months after treatment with I131 and thereafter as needed (in patients treated with I131).

### Statistical analysis

SPSS software version 26.0 was used for data analysis. Measured data are expressed as mean ± standard deviation (SD), median and interquartile range (IQR), and count data are expressed as weighted frequency and percentage (%). Continuous variables were analysed using the T-test or the Kruskal-Wallis test in the case of a non-normal distribution. Categorical variables were analysed using the Chi-square test or the Kruskal-Wallis test. For correlation analysis, Spearman rank correlation analysis was used and survival curves were constructed using the Kaplan-Meier method. For regression analysis, a multivariate linear regression model was used. If P < was 0.05, the difference was considered statistically significant.

## Observations and results

### Characteristics of included patients

147 patients with MTC who underwent surgery were included in the study. After inclusion and exclusion, 30 cases of synchronous MTC/PTC (combination group) and 92 cases of simple MTC (simple group) were finally included. The follow-up period lasted until 28 February 2023 (i.e. the median follow-up time was 81.3 months).

### Baseline comparison

Compared with the simple group, the combination group had a higher proportion of men (56.7% vs 33.7%, P=0.025), a lower preoperative Ctn score [29.9 (2.8, 201.6) vs 199.9 (25.4, 585.0), P=0.001], a lower preoperative CEA score [5.7 (4.1, 22.5) vs 17.5 (6.4, 54.7), P=0.048] and a lower proportion of MTC lesions with calcification (36.7% vs 67.4%, P=0.003). There was no statistically significant difference between the two groups in age, BMI, US echo of the MTC lesion, margin and blood flow signal (P > 0.05), as shown in **Table 1**.

**Table 1:** Comparison of Clinical Baseline Data between Combined Group and Simple Group [Cases (%)].

Variable	Combined Group	Simple Group	Z/χ2	P-value
Gender (n)			5.002	0.025
Male	17(56.7)	31(33.7)		
Female	13(43.3)	61(66.3)		
Age (years)			2.105	0.147
≤55	18(60)	68(73.9)		
>55	12(40)	24(26.1)		
BMI(kg/m^2^)			0.033	0.855
≤24	21(70)	66(71.7)		
>24	9 (30)	26(28.3)		
Preoperative Ctn (Pg/mL)	29.9(2.8,201.6)	199.9(25.4,585.0)	-3.304	0.001
Preoperative CEA (ng/mL)	5.7(4.1,22.5)	17.5(6.4,54.7)	-1.981	0.048
Ultrasound Echo (n)			1.180	0.277
Low Echo	18(60)	65(70.7)		
Other Echo	12(40)	27(29.3)		
Boundary (n)			0.696	0.404
Clear	15(50)	38(41.3)		
Unclear	15(50)	54(58.7)		
Calcification (n)			8.886	0.003
Present	11(36.7)	62(67.4)		
Absent	19(63.3)	30(32.6)		
Blood Flow Signal (n)			1.337	0.248
Present	15(50)	57(61.9)		
Absent	15(50)	35(38.1)		

### Comparison of surgical procedures

Compared with the simple group, a higher proportion of patients in the combined group underwent bilateral total thyroidectomy with central lymph node dissection (46.7% versus 20.7%, P=0.034). There was no statistically significant difference between the two groups in terms of operative time, postoperative hospital stay and occurrence of complications [recurrent laryngeal nerve damage, hypoparathyroidism (hypocalcaemia), superior laryngeal nerve damage, lymph fistula, incisional haematoma, incisional infection, thyroid crisis, etc.] (P > 0.05), as shown in **Table 2**.

**Table 2:** Comparison of Surgical Data between Combined Group and Simple Group [Cases (%)].

Variable	Combined Group	Simple Group	t/χ2	P-value
Surgical Method (n) UT+CLND TT+CLND TT+CLND+ULND TT+CLND+BLND			8.643	0.034
	3 (10)	7 (7.6)		
	14 (46.7)	19 (20.7)		
	10 (33.3)	50 (54.3)		
	3 (10)	16 (17.4)		
Surgery Time (h)	3.14±2.15	3.35±1.05	-0.299	0.767
Postoperative Hospital Stay (d)	4.5±1.9	4.9±1.9	-1.512	0.132
Complications (n) Present Absent			0.006	0.939
	6 (20)	19 (20.7)		
	24 (80)	73 (79.3)		

UT, Unilateral thyroidectomy; TT, Total thyroidectomy; CLND, Central lymph node dissections.

ULND, Unilateral neck lymph node dissection; BLND, Bilateral neck lymph node dissection.

### Pathology

Compared with the simple group, patients in the combined group had MTC lesions that were exclusively single foci (100% vs. 78.3%, P = 0.005) and a lower proportion of combined thyroid nodules (53.3% vs. 84.8%, P < 0.001), a higher proportion of combined thyroiditis (23.3% vs. 8.7%, P = 0.034) and a lower mean number of lymph nodes removed [9.5 (2.3, 22.5) vs. 21.0 (7.0, 34.0), P = 0.011]. There was no statistically significant difference between the two groups in terms of lesion location, lesion diameter, extracapsular invasion, mean number of lymph node metastases, TNM stage and mean percentage of Ki67 (P > 0.05), as shown in **Table 3**.

**Table 3:** Comparison of MTC pathological results between combined group and simple group [cases (%)].

Variable	Combined Group	Simple Group	Z/χ2	P-value
Lesion location (n)			4.472	0.107
Left lobe	15(50)	43(46.7)		
Right lobe	15(50)	37(40.2)		
Both lobes	0	12(13.1)		
Lesion diameter (cm)	0.8(0.4,2.0)	1.4(0.7,2.4)	-1.669	0.095
Number of lesions (n)			7.801	0.005
Single lesion	30(100)	72(78.3)		
Multiple lesions	0	20(21.7)		
Extrathyroidal extension (n)			0.268	0.605
Yes	2 (6.7)	9 (9.8)		
No	28(93.3)	83(90.2)		
Combined nodular goiter(n)			12.653	<0.001
Yes	16(53.3)	78(84.8)		
No	14(46.7)	14(15.2)		
Combined thyroiditis (n)			4.495	0.034
Yes	7 (23.3)	8 (8.7)		
No	23(76.7)	84(91.3)		
CLN dissection number	2(4,8)	6(4,10)	-1.692	0.091
CLN metastasis number	1(2,2)	3(2,6)	-1.596	0.110
LLN dissection number	13.5(19.5,31.5)	15(19,28)	-0.039	0.969
LLN metastasis number	5(1.5,19.5)	4.5(2,9)	-0.200	0.841
T stage (n)			0.715	0.398
T1, T2	27(90)	77(83.7)		
T3, T4	3(10)	15(16.3)		
N stage (n)			3.638	0.162
N0	15(50)	39(42.4)		
N1a	7 (23.3)	12(13.1)		
N1b	8 (26.7)	41(44.5)		
M stage (n)			2.936	0.087
M0	28(93.3)	91(98.9)		
M1	2 (6.7)	1 (1.1)		
Ki67 (%)	1.0(1.0,4.0)	2.0(1.0,5.0)	-0.760	0.447

P value, Statistical significance value; T stage, Tumor stage; N stage, Lymph node stage; M stage, Metastasis stage; Ki67, A protein that serves as a cell proliferation marker; CLN, Central lymph node; LLN, Lateral lymph node.

The combined group was divided into two groups according to whether the diameter of the MTC lesion was greater than 1 cm. Preoperative calcitonin, preoperative CEA, extracapsular invasion, the presence of lymph node metastases, stage and Ki67 were included in the analysis. The results showed that when MTC lesion diameter was ≤1 cm, preoperative CEA was lower in the combined group than in the plain group [4.7 (2.1, 5.4) vs. 5.5 (4.8, 18.4), P = 0.032]; the differences in the other aspects were not statistically significant (P > 0.05), as shown in **Table 4**. When the MTC lesion diameter of the two groups > was 1 cm, the preoperative calcitonin level of the combined group was lower compared with the simple group [206.4 (61.7, 585.0) vs 491.5 (171.8, 833.5), P = 0.021]; the differences in other aspects were not statistically significant (P > 0.05), as shown in **Table 4**.

**Table 4:** Comparison of malignancy degree between the combined group and the simple group [cases (%)].

	Tumor Diameter ≤ 1cm				Tumor Diameter > 1cm			
	Combined Group	Simple Group	Z/χ2	P value	Combined Group	Simple Group	Z/χ2	P value
Preoperative Calcitonin (pg/mL)	13.1 (2.5,36.2)	21.3 (6.4,112.8)	-1.253	0.210	206.4 (61.7,585.0)	491.5 (171.8,833.5)	-2.305	0.021
Preoperative CEA (ng/mL)	4.7 (2.1,5.4)	5.5 (4.8,18.4)	-2.141	0.032	26.3 (13.1,198.3)	25.5 (13.0,67.8)	-0.020	0.984
Extrathyroidal extension (n)			1.038	0.308			0.641	0.423
Yes	0	2 (5.6)			2 (16.7)	5 (8.9)		
No	18 (100)	34 (94.4)			10 (83.3)	51 (91.1)		
Lymph Node Metastasis (n)			0.153	0.695			0.578	0.447
Yes	8 (44.4)	14 (38.9)			7 (58.3)	39 (69.6)		
No	10 (55.6)	22 (61.1)			5 (41.7)	17 (30.4)		
Stage (n)			0.153	0.695			1.054	0.305
I, II	10 (55.6)	22 (61.1)			5 (41.7)	15 (26.8)		
III, IV	8 (44.4)	14 (38.9)			7 (58.3)	41 (73.2)		
Ki67 (%)	1.0 (1.0,3.5)	1.0 (1.0,3.5)	-0.262	0.794	1.0 (1.0,5.0)	3.0 (1.0,5.0)	-0.795	0.427

### Compare the subtypes of synchronous MTC/PTC and simple MTC

Compare the clinical data of type III and type IV in the combined group with those of the simple group. Due to the small number of type I and type II cases in the combined group, they were not included in this comparison. Compared to the simple group, the type III had lower preoperative Ctn values [23.2(2,5,144.3) vs. 199.9(25,4,585.0), P=0.005], lower preoperative CEA values [4.9(2,12.6) vs. 17.5(6,4,54.7), P=0.017]. There was no statistically significant difference between the type III and plain group patients in terms of sex, age, BMI, MTC lesion echo, boundary and blood flow signal (P > 0.05). Type IV had a higher proportion of males (70% vs 33.7%, P=0.024), a lower proportion of low echo MTC lesions (40% vs 70.7%, P=0.049). Type III and type IV had a lower proportion of calcified MTC lesions (28.6% vs 67.4%, P=0.005), (30% vs 67.4%, P=0.02). There was no statistically significant difference between the type IV and simple group patients in terms of age, BMI, preoperative Ctn, preoperative CEA, MTC lesion border and blood flow signal (P > 0.05), see **Table 5**.

**Table 5:** Comparison of Clinical Baseline Data between Type III, Type IV and Simple Group [Cases (%)].

Variable	Simple Group	Type III	Type IV	Z/χ2		P-value	
				Type III	Type IV	Type III	Type IV
Gender (n)				1.405	5.086	0.236	0.024
Male	31 (33.7)	7 (50)	7 (70)				
Female	61 (66.3)	7 (50)	3 (30)				
Age (years)				0.567	0.877	0.452	0.349
≤55	68 (73.9)	9 (64.3)	6 (60)				
>55	24 (26.1)	5 (35.7)	4 (40)				
BMI (kg/m^2^)				1.221	0.013	0.269	0.908
≤24	66 (71.7)	12 (85.7)	7 (70)				
>24	26 (28.3)	2 (14.3)	3 (30)				
Preoperative Ctn (Pg/mL)	199.9 (25.4,585.0)	23.2 (2.5,144.3)	202.8 (3.6,585)	-2.788	-0.792	0.005	0.429
Preoperative CEA (ng/mL)	17.5 (6.4,54.7)	4.9 (2,12.6)	75.7 (2.9,303.8)	-2.384	-0.033	0.017	0.974
Ultrasound Echo (n)				0.234	3.872	0.629	0.049
Low Echo	65 (70.7)	9 (64.3)	4 (40)				
Other Echo	27 (29.3)	5 (35.7)	6 (60)				
Boundary (n)				1.241	0.006	0.265	0.937
Clear	38 (41.3)	8 (57.1)	4 (40)				
Unclear	54 (58.7)	6 (42.9)	6 (60)				
Calcification (n)				7.793	5.455	0.005	0.02
Present	62 (67.4)	4 (28.6)	3 (30)				
Absent	30 (32.6)	10 (71.4)	7 (70)				
Blood Flow Signal (n)				0.726	0.541	0.394	0.462
Present	57 (61.9)	7 (50)	5 (50)				
Absent	35 (38.1)	7 (50)	5 (50)				

Compared with the plain group, type III and type IV had a lower proportion of nodal goitre (42.9% vs. 84.8%, P < 0.001), (50% vs. 84.8%, P=0.007), type III had a higher proportion of thyroiditis (28.6% vs. 8.7%, P=0.029), fewer central lymph node dissections [3(2,5) vs. 6(4,10), P=0.033], fewer central lymph node metastases [0(0,0) vs 3(2,6), P=0.005], fewer lateral neck lymph node metastases [0(0,0,5) vs 4,5(2.9), P=0.035], higher proportion of N0 stage (78.6% vs 42.4%, P=0.012), lower Ki67 score [1.0(1.0,1.0) vs 2.0(1.0,5.0), P=0.032]. There was no statistically significant difference between the III and plain group patients in terms of operative time, postoperative hospital stay, postoperative complications, lesion location, lesion diameter, extrathyroidal invasion, lateral cervical lymph node dissection, T stage and M stage (P > 0.05), see **Table 6**. There was no statistically significant difference between the IV type patients and the simple group in terms of operative time, postoperative hospital stay, postoperative complications, lesion location, lesion diameter, extrathyroidal invasion, thyroiditis, central lymph node dissection, central lymph node metastasis, lateral cervical lymph node dissection, lateral cervical lymph node metastasis, T stage, N stage, M stage and Ki67 (P > 0.05), see **Table 6**.

**Table 6:** Comparison of Surgical Data and MTC pathological results between Type III, Type IV and Simple Group [Cases (%)].

Variable	Simple Group	Type III	Type IV	*t/Z/χ2*		P-value	
				Type III	Type IV	Type III	Type IV
Surgery Time (h)	3.35±1.05	3.18±1.26	3.1±1.45	0.553	0.682	0.582	0.497
Postoperative Hospital Stay (d)	4.9±1.9	4.4±1.8	4.7±1.3	1.18	0.569	0.241	0.571
Complications (n)				1.449	0.002	0.229	0.961
Present	19 (20.7)	1 (7.2)	2 (20)				
Absent	73 (79.3)	13 (92.8)	8 (80)				
Lesion location (n)				0.055	0.674	0.814	0.412
Left lobe	43 (46.7)	8 (57.1)	4 (40)				
Right lobe	37 (40.2)	6 (42.9)	6 (60)				
Both lobes	12 (13.1)	0	0				
Lesion diameter (cm)	1.4 (0.7,2.4)	0.8 (0.5,1.8)	0.8 (0.6,2.5)	-1.577	-0.792	0.115	0.428
Extrathyroidal extension (n)				1.497	0.001	0.221	0.982
Yes	9 (9.8)	0	1 (10)				
No	83 (90.2)	14 (100)	9 (90)				
Combined nodular goiter (n)				12.986	7.199	<0.001	0.007
Yes	78 (84.8)	6 (42.9)	5 (50)				
No	14 (15.2)	8 (57.1)	5 (50)				
Combined thyroiditis (n)				4.781	1.303	0.029	0.254
Yes	8 (8.7)	4 (28.6)	2 (20)				
No	84 (91.3)	10 (71.4)	8 (80)				
CLN dissection number	6 (4,10)	3 (2,5)	4.5 (2,10.5)	-2.127	-0.591	0.033	0.555
CLN metastasis number	3 (2,6)	0 (0,0)	1 (0,1.25)	-2.787	-0.56	0.005	0.576
LLN dissection number	15 (19,28)	19 (16,22)	17.5 (12,32)	-0.252	-0.273	0.801	0.785
LLN metastasis number	4.5 (2,9)	0 (0,0.5)	2 (0.8,6)	-2.105	-0.388	0.035	0.698
T stage (n)				2.659	0.271	0.103	0.603
T1, T2	77 (83.7)	14 (100)	9 (90)				
T3, T4	15 (16.3)	0	1 (10)				
N stage (n)				6.383	0.572	0.012	0.45
N0	39 (42.4)	11 (78.6)	3 (30)				
N1	53 (57.6)	3 (21.4)	7 (70)				
M stage (n)				0.154	3.727	0.695	0.054
M0	91 (98.9)	14 (100)	9 (90)				
M1	1 (1.1)	0	1 (10)				
Ki67 (%)	2.0 (1.0,5.0)	1.0 (1.0,1.0)	3.0 (1.0,7.5)	-2.138	-0.387	0.032	0.699

P value, Statistical significance value; T stage, Tumor stage; N stage, Lymph node stage; M stage, Metastasis stage; Ki67, A protein that serves as a cell proliferation marker; CLN, Central lymph node; LLN, Lateral lymph node.

### Correlation between preoperative serum Ctn and clinicopathological features of the two patient groups

Of the 92 patients in the simple group, 74 patients (80.4%) had elevated preoperative serum Ctn values; 18 patients (19.6%) had normal values. The results of univariate analysis showed that patients aged > 55 years had higher preoperative serum Ctn values compared with those aged ≤ 55 years (P < 0.001); patients with larger tumour diameters had higher preoperative serum Ctn values (P < 0.001), which had a positive correlation of 23.20 Pg/ml, 193.00 Pg/ml, 585.10 Pg/ml and 585.00 Pg/ml, respectively; compared with patients without combined lymphocytic thyroiditis, patients with combined lymphocytic thyroiditis had higher preoperative serum Ctn levels (P = 0.038). No significant association was found between sex (P = 0.065), BMI (P = 0.122), number of MTC lesions (P = 0.068), tumour distribution (P = 0.178), extracapsular invasion (P = 0.212), N stage (P = 0.a multivariate regression model was constructed (F = 15.996, P < 0.01), which showed that the number of MTC lesions and maximum tumour diameter correlated with preoperative serum Ctn level.

Of the 30 patients in the combined group, 21 (70%) had elevated preoperative serum Ctn levels; 9 (30%) were within the normal range. Univariate analysis showed that patients with larger tumour diameters had higher preoperative serum Ctn levels (P=0.025), indicating a positive correlation, which was 17.43 Pg/ml, 92.20 Pg/ml, 347.00 Pg/ml and 585.00 Pg/ml, respectively. No significant association was found between preoperative Ctn serum levels and sex (P=0.199), age (P=0.216), BMI (P=0.882), tumour distribution (P=0.289), N staging (P=0.149), presence of nodular thyroid goitre (P=0.720) and presence of lymphocytic thyroiditis (P=0.648). A multivariate regression model was constructed (F=1.113, P=0.042), which showed that age was correlated with preoperative serum Ctn levels.

Preoperative serum Ctn correlated with maximum tumour diameter in both patient groups using Spearman correlation analysis (see **Table 7**). In the patients in the pure group, preoperative serum Ctn was positively correlated with maximum tumour diameter with a correlation coefficient of 0.688 (P < 0.001). In patients in the combined group, preoperative serum Ctn was positively correlated with maximum tumour diameter, with a correlation coefficient of 0.463 (P=0.008).

**Table 7:** Analysis of the correlation between preoperative serum Ctn and tumor maximum diameter.

Numbers			Preoperative Ctn	P value
			Correlation Coefficient	
Tumor Diameter	Simple Group	92	0.688	<0.001
	Combined Group	30	0.463	0.008

As shown in **Table 8**, the preoperative serum Ctn value correlated with the situation of lymph node metastases in both patient groups by Spearman correlation analysis. In the pure group, the maximum diameter of the metastatic lesion, the total number of lymph node metastases, the number of ipsilateral cervical lymph node metastases and the rate of ipsilateral cervical lymph node metastases were positively correlated with preoperative serum Ctn, with correlation coefficients of 0.471 (P=0.001), 0.289 (P=0.049), 0.281 (P=0.026) and 0.363 (P=0.013), respectively. No association was found between preoperative serum Ctn and lymph node metastases in the combined patient group.

**Table 8:** Analysis of the correlation between preoperative serum Ctn and lymph node metastasis status.

	Ctn	Numbers	Correlation Coefficient	P value
Simple Group	Maximum Metastatic Focus Diameter	47	0.471	0.001
	Total LN Metastasis Count	47	0.289	0.049
	Total LN Metastasis Rate	47	0.108	0.469
	Central LN Metastasis Count	43	0.045	0.768
	Central LN Metastasis Rate	43	0.167	0.272
	Ipsilateral Cervical LN Metastasis Count	35	0.281	0.026
	Ipsilateral Cervical LN Metastasis Rate	35	0.363	0.013
Combined Group	Maximum Metastatic Focus Diameter	16	0.263	0.326
	Total LN Metastasis Count	16	0.082	0.762
	Total LN Metastasis Rate	16	-0.192	0.475
	Central LN Metastasis Count	15	-0.315	0.252
	Central LN Metastasis Rate	15	0.023	0.934
	Ipsilateral Cervical LN Metastasis Count	8	-0.393	0.336
	Ipsilateral Cervical LN Metastasis Rate	8	-0.252	0.548

LN, lymph node.

**Figure 1:**
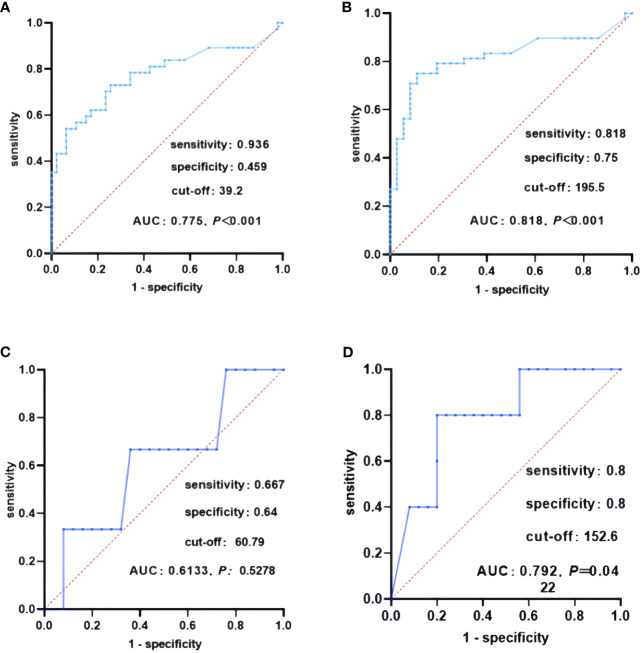
ROC curves of lymph node metastasis in both patient groups. **(A)** ROC curves of lymph node metastasis in the pure group, **(B)** ROC curves of lateral lymph node metastasis in the pure group, **(C)** ROC curves of lymph node metastasis in the combined group, **(D)** ROC curves of lateral lymph node metastasis in the combined group.

Subsequently, this study investigated the influence of preoperative serum Ctn on the situation of lymph node metastases in the two patient groups by creating ROC curves. The results showed that for patients in the pure group, the cut-off value of preoperative serum Ctn for the occurrence of cervical lymph node metastases was 39.2pg/ml, the area under the curve (AUC) was 0.775, the sensitivity was 93.the sensitivity was 93.6% and the specificity was 45.9% (**Figure 1A**); the preoperative Ctn cut-off for the presence of lateral cervical lymph node metastases was 195.5pg/ml, the AUC was 0.818, the sensitivity was 81.8% and the specificity was 75% (**Figure 1B**). For the patients in the combined group, the cut-off value of preoperative serum Ctn for the presence of cervical lymph node metastases was 60.79pg/ml, AUC was 0.613, sensitivity was 66.7% and specificity was 64% (**Figure 1C**), Ctn for the presence of lateral cervical lymph node metastases was 152.6pg/ml, AUC was 0.792, sensitivity was 80% and specificity was 80% (**Figure 1D**).

**Figure 2:**
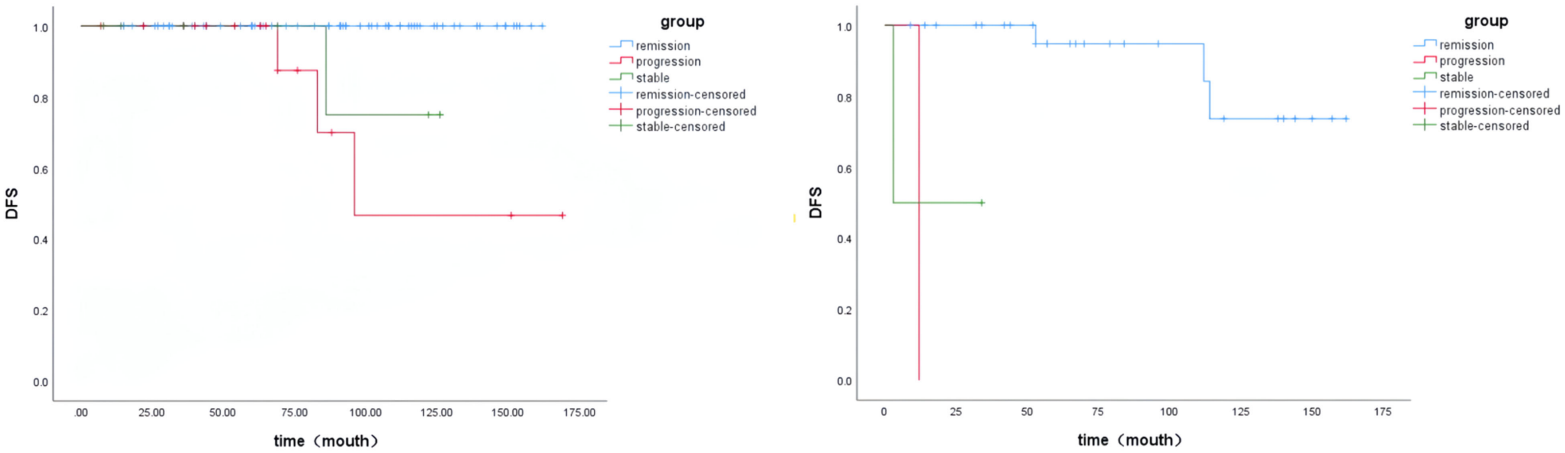
KM curves of both patient groups.

**Figure 3:**
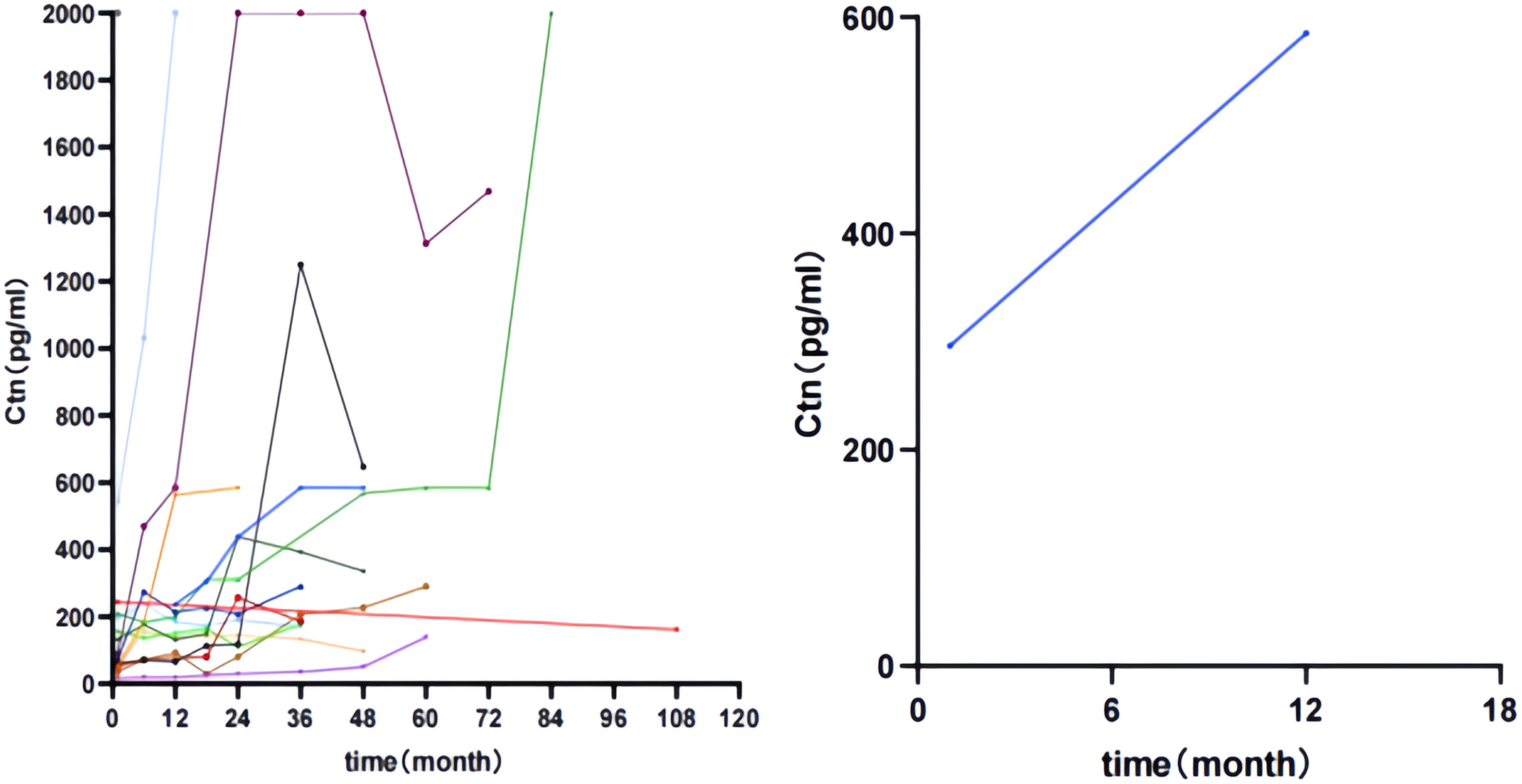
Change curves of postoperative serum Ctn in the progression group of both patient groups.

Patients in the two groups were divided into three groups based on postoperative serum Ctn values. Remission group: postoperative serum Ctn value decreased to normal and remained stable; Stability group: postoperative serum Ctn value did not decrease to normal but remained stable; Progression group: postoperative serum Ctn value was above 150 Pg/ml or the doubling time was less than 12 months. The K-M curves were plotted according to the disease-free survival (DFS) of the patients. As shown in **Figure 2**, the prognosis of the progression group was significantly worse in the pure group (P < 0.001). As shown in **Figure 3**, Compared to the simple group, the combined group had a lower proportion in the progression group but a faster postoperative Ctn progression.

### Correlation between preoperative CEA and clinicopathological features of the two patient groups

Of the 92 patients in the pure group, 50 were tested preoperatively for CEA, and 40 (80%) had elevated preoperative CEA levels; 10 (20%) had normal levels. Univariate analysis showed that patients with larger tumour diameters had higher preoperative CEA levels (P < 0.001), indicating a positive correlation, which were 5.7ng/ml, 20.6ng/ml, 26.5ng/ml and 499.8ng/ml, respectively. No significant correlation was found between preoperative CEA levels and gender (P=0.921), age (P=0.086), BMI (P=0.992), number of MTC lesions (P=0.117), tumour distribution (P=0,056), extracapsular invasion (P=0.792), N staging (P=0.149), presence of nodular thyroid goitre (P=0.252) and presence of lymphocytic thyroiditis (P=0.8) were found. A multivariate regression model was constructed (F=3.373, P=0.002), which showed that age and tumour diameter ≥4 cm correlated with preoperative CEA levels.

Of the 15 patients in the combined group who had preoperative CEA testing, 9 (60%) had elevated preoperative CEA levels; 6 (40%) were within the normal range. Univariate analysis showed that patients with larger tumour diameters had higher preoperative CEA levels (P=0.024), indicating a positive correlation, with values of 4.7ng/ml, 22.6ng/ml, 145.5ng/ml and 356.5ng/ml, respectively. Compared to females, male patients had higher preoperative CEA levels (P=0.015); compared to patients aged ≤55 years, patients aged > 55 years had higher preoperative CEA levels (P=0.046). No significant association was found between preoperative CEA levels and BMI (P=0.306), tumour distribution (P=0.668) and presence of nodular thyroid goitre (P=0.886). After building a multivariate regression model, no correlation was found with preoperative CEA levels.

A Spearman correlation analysis was performed between the preoperative CEA value and the maximum tumour diameter of the patients in both groups (see **Table 9**). In the pure group, the preoperative CEA value was positively correlated with the maximum tumour diameter, with a correlation coefficient of 0.491 (P < 0.001). In the combined group, the preoperative CEA value was positively correlated with the maximum tumour diameter, with a correlation coefficient of 0.993 (P < 0.001).

**Table 9:** Correlation analysis between preoperative CEA and tumor maximum diameter.

Numbers			Preoperative CEA	P-value
			Correlation Coefficient	
Tumor Diamete	Simple Group	50	0.491	<0.001
	Correlation Coefficient	15	0.933	<0.001

As shown in **Table 10**, a Spearman correlation analysis was performed between the preoperative CEA value and the lymph node metastasis status of the patients in both groups. In the pure group, the maximum diameter of metastatic lesions, the total number of lymph node metastases, the number of ipsilateral cervical lymph node metastases and the rate of ipsilateral cervical lymph node metastases were positively correlated with preoperative CEA, with correlation coefficients of 0.573 (P < 0.001), 0.424 (P=0.014), 0.457 (P=0.017) and 0,489 (P=0,004). No association was found between the preoperative CEA value and the lymph node metastasis status in the combined group.

**Table 10:** Correlation analysis of preoperative serum CEA level and lymph node metastasis status.

	CEA	Numbers	Correlation Coefficient	P-value
Simple Group	Maximum Metastatic Focus Diameter	33	0.573	<0.001
	Total LN Metastasis Count	33	0.424	0.014
	Total LN Metastasis Rate	33	0.084	0.641
	Central LN Metastasis Count	32	0.128	0.479
	Central LN Metastasis Rate	32	0.122	0.499
	Ipsilateral Cervical LN Metastasis Count	24	0.457	0.017
	Ipsilateral Cervical LN Metastasis Rate	24	0.489	0.004
Combined Group	Maximum Metastatic Focus Diameter	7	0.607	0.148
	Total LN Metastasis Count	7	0.741	0.057
	Total LN Metastasis Rate	7	0.487	0.268
	Central LN Metastasis Count	7	0.49	0.264
	Central LN Metastasis Rate	7	0.595	0.159
	Ipsilateral Cervical LN Metastasis Count	4	-0.2	0.800
	Ipsilateral Cervical LN Metastasis Rate	4	0.4	0.600

LN, lymph node.

### Follow-up

There was no statistically significant difference between the two groups in terms of follow-up time, calcitonin one month after surgery, calcitonin at last follow-up, CEA one month after surgery and CEA at last follow-up (P > 0.05), as shown in **Table 11**.

**Table 11:** Comparison of follow-up data of the two groups of patients[Cases (%)].

Variable	Combined Group	Simple Group	*t/Z*	P-value
Follow-up Duration (Months)	73.7±47.7	83.7±43.3	-1.154	0.250
Calcitonin One Month After Surgery (pg/mL)	1.5(0.6,7.1)	8.6(0.6,82.6)	-1.942	0.052
Calcitonin at the latest follow-up(pg/mL)	1.0(0.5,58.8)	6.7(0.6,98.9)	-0.913	0.361
CEA One Month After Surgery (ng/mL)	4.5(2.1,39.7)	9.1(2.9,21.4)	-0.552	0.581
CEA at the latest follow-up(ng/mL)	3.1(1.8,5.3)	3.1(1.6,9.6)	-0.047	0.963

## Discussion

The co-occurrence of PTC and MTC is rare and accounts for less than 1% of thyroid tumours according to the literature (2–6). In 14 years, 28,621 thyroid tumours were operated in our centre. Among these 28,621 thyroid tumour cases, only 30 cases of co-occurrence of medullary and papillary thyroid carcinomas were found, which corresponds to an incidence rate of 0.1%. In previous studies at our centre, the 30 patients with co-occurrence of medullary and papillary thyroid carcinomas were divided into four categories based on the location of the tumours (2). The pathogenesis of synchronous medullary and papillary thyroid carcinoma is a much debated topic among scientists at home and abroad, with theories such as the “hijacking” hypothesis, the local effect hypothesis, the stem cell hypothesis, the differentiation hypothesis and the collision hypothesis (11–15). In this study, the type I and II patients support the “hijacking” hypothesis, while the type III and IV patients mainly support the collision hypothesis. However, none of these theories can fully explain the pathogenesis of the disease, and there is not enough evidence or theory to support or rule out any hypothesis.

Regarding the basic clinical data, some researchers have pointed out that the average age of the combined group is higher than that of the pure group, but some researchers believe that the age difference between the two groups is not statistically significant (11–15). This study found that the age difference between the combined group and the pure group was not statistically significant (P=0.147). This study found that the combined group had a higher incidence in males, lower preoperative Ctn values and lower preoperative CEA values compared to the pure group. Regarding ultrasound features, the proportion of MTC lesions with calcification was lower in the combined group. This suggests that the presence of PTC in the combined group might influence the secretion of Ctn and CEA by MTC, which requires careful preoperative examination to avoid omission. There was no statistically significant difference between the two groups in other basic clinical data.

Regarding surgical data, this study found that the combined group had a higher proportion of bilateral total thyroidectomy + central lymph node dissection compared to the simple group. The simple group had a higher proportion of bilateral total thyroidectomy + ipsilateral/bilateral central neck lymph node dissection. There were no differences between the two groups in terms of removal and metastasis of central and lateral neck lymph nodes. The extent of surgery in the simple group was more aggressive and the extent of removal was greater than in the combined group, but there was no difference in lymph node removal and metastasis between the two groups. Both groups removed the lymph nodes thoroughly and systematically. There was no statistical significance in other surgical data between the two groups (P>0.05).

From a pathological point of view, some researchers pointed out that the maximum diameter of the MTC was smaller in the combined group than in the simple group, so that more careful dissection should be performed during pathological examination to avoid missing PTC lesions; however, other researchers consider that the difference between the two groups in the maximum diameter of the MTC is not statistically significant. In this study, the maximum diameter of the MTC was smaller in the combined group than in the simple group [0.8 (0.4, 2.0) vs. 1.4 (0.7, 2.4), P=0.095], but no statistical difference was observed. Compared to the simple group, all MTC lesions in the combined group were single lesions, the proportion of nodular goitre was lower and the proportion of thyroiditis was higher. This suggests that the occurrence of PTC in the combined group may be related to a higher incidence of thyroiditis and a lower incidence of nodular goitre compared to the simple group. It is possible that these are coincidental single lesions. It is worth noting that in the combined group, PTC occurred in 9 patients and MTC in 4 patients, and medullary and papillary thyroid carcinomas occurred simultaneously in 2 patients. There was no statistical significance between the two groups in other pathological data. To investigate whether there was a difference between the two groups when the diameter of the MTC was the same, the two groups were divided according to the diameter of the MTC lesions. When the diameter of the MTC lesions in the two groups was ≤1cm, the preoperative CEA level of the combined group was lower compared to the untreated group and the preoperative calcitonin level was not statistically significant; when the diameter of the MTC lesions in the two groups was < 1cm, the preoperative calcitonin level of the combined group was lower compared to the untreated group; the preoperative CEA level was not statistically significant.

In line with our previous studies, we divided the combined group into different subtypes and compared them with the clinical data of the simple group. Due to the small number of cases in type I and type II, only type III and type IV were included in this comparison. Compared to the plain group, type III had lower preoperative Ctn values, lower preoperative CEA values, lower proportion of calcified MTC lesions, lower proportion of nodular goitre, higher proportion of thyroiditis, fewer central lymph node dissections, fewer central lymph node metastases, fewer lateral neck lymph node metastases, higher proportion of N0 stage and lower Ki67. Compared to the untreated group, the IV type had a higher proportion of males, a lower proportion of echo-deficient MTC lesions, a lower proportion of calcified MTC lesions and a lower proportion of nodular goitre. The III type was significantly different from the simple group, while the IV type was more similar to the simple group. This could be due to the fact that the MTC and PTC lesions in the IV type were further apart and influenced each other less, but were more similar to the simple MTC.

Ctn and CEA are important for MTC. This study investigated the correlation between Ctn and CEA and clinicopathological features in two groups of patients. In univariate analysis, the preoperative Ctn values of both patient groups were positively correlated with MTC diameter. However, in the simple group, preoperative Ctn values were associated with age and the presence of thyroiditis, whereas this phenomenon was not observed in the combined group. In multivariate analysis, preoperative Ctn values in the simple group were associated with the number of MTC lesions and maximum tumour diameter, while preoperative Ctn values in the combined group were related to age. Spearman correlation analysis to investigate the strength of the association between preoperative Ctn values and MTC diameter in the two patient groups showed that preoperative Ctn values in the simple group were strongly correlated with MTC diameter, while preoperative Ctn values in the combined group were moderately correlated with MTC diameter. Next, we analysed the correlation between preoperative Ctn values and lymph node metastases in two patient groups. This study found that the preoperative serum Ctn level in the simple group was positively correlated with the maximum diameter of metastatic lesions, the total number of lymph node metastases, the number of ipsilateral cervical lymph node metastases and the ipsilateral cervical lymph node metastasis rate, while no correlation was found between preoperative Ctn level and lymph node metastases in the combined group. When ROC curves were plotted for two groups of patients in this study, it was found that the combined group had a higher Ctn level when lymph node metastases occurred, but a lower Ctn level when ipsilateral cervical lymph node metastases occurred than the pure group. KM curves were generated using the patients’ postoperative serum Ctn levels. This study showed that the prognosis of both groups of patients was significantly worse in the progression group. In the simple group, there was no statistical difference between the remission group and the stable group, while in the combined group, the prognosis of the stable group was significantly worse than that of the remission group, and there was no statistical difference between the progression group and the stable group. This suggests that patients in the combined group are more likely to relapse when postoperative Ctn levels are stable or below 150pg/ml than patients in the simple group. Compared to the simple group, the combined group had a lower proportion in the progression group but a faster postoperative Ctn progression.

In univariate analysis, CEA levels in the simple group were positively correlated with tumour diameter, whereas CEA levels in the combined group were correlated with tumour diameter, age and sex. In multivariate analysis, CEA levels in the simple patient group were associated with age and tumour diameter ≥4 cm, while no correlation with CEA levels was found in the combined patient group. Spearman correlation analysis to examine the strength of correlation between CEA levels and MTC diameter in two patient groups showed that CEA levels in the simple group were moderately correlated with MTC diameter, while CEA levels in the combined group were strongly correlated with MTC diameter. This may suggest that CEA levels in the combined group of MTC have better predictive power for tumour recurrence than in the simple group. Next, we analysed the correlation between CEA levels and lymph node metastases in two groups. This study found that CEA levels in the simple group were positively correlated with the maximum diameter of metastatic lesions, the total number of lymph node metastases, the number of ipsilateral cervical lymph node metastases and the ipsilateral cervical lymph node metastasis rate, while no correlation was found between CEA levels and lymph node metastases in the combined group.

In terms of follow-up data, some investigators have pointed out that there is no statistical difference between the follow-up data of the simple group and the combined group. The present study supports this view and found no difference in follow-up between the two groups of patients.

This study is currently probably the largest comparative analysis study of synchronous medullary and papillary thyroid carcinoma and MTC performed to date and compares for the first time the correlation between Ctn and CEA and clinical case characteristics of two groups of patients. In synchronous medullary and papillary thyroid carcinomas, there is not only a simple co-occurrence of MTC lesions and PTC lesions, but also a collision of the biological features of two tumours. The PTC affects the MTC secretion of Ctn and CEA, lymph node metastasis and the extent of surgical intervention to some extent, which in turn affects the prognosis of patients. Although 30 patients in the combined group and 92 patients in the simple group were enrolled in this study, which is more than previous case reports and clinical trials, the total number of cases is still small. The study needs further confirmation of our findings because: (i) the small number of these rare diseases (MTC/PTC, 1% prevalence), (ii) the associated lack of expertise and knowledge about MTC/PTC (among endocrinologists as well as surgeons and pathologists) pose a major challenge for physicians. There are only a few surgeons/endocrinologists/pathologists dealing with patients with this rare disease and they usually work in a limited number of specialised centres. De facto, a large multicentre study is needed, involving mainly high volume centres with a dedicated endocrine pathologist, with interdisciplinary exchange and appropriate time frame.

## Data availability statement

The original contributions presented in the study are included in the article/supplementary material. Further inquiries can be directed to the corresponding author.

## Ethics statement

The study was approved by the China-Japan Union Hospital of Jilin University ethics committee (No. 20220804013). As this was a retrospective study, informed consent was waived. The studies were conducted in accordance with the local legislation and institutional requirements. The participants provided their written informed consent to participate in this study.

## Author contributions

DZ: Writing – original draft, Writing – review & editing. MY: Writing – original draft. FF: Writing – original draft, Writing – review & editing. AC: Writing – original draft. KL: Writing – original draft. HW: Writing – original draft. HC: Writing – original draft. CS: Writing – original draft. KB: Writing – original draft. DL: Writing – original draft. GD: Writing – original draft, Writing – review & editing. HS: Writing – original draft, Writing – review & editing.
